# Falling Weight Impact Damage Characterisation of Flax and Flax Basalt Vinyl Ester Hybrid Composites

**DOI:** 10.3390/polym12040806

**Published:** 2020-04-03

**Authors:** Hom Nath Dhakal, Elwan Le Méner, Marc Feldner, Chulin Jiang, Zhongyi Zhang

**Affiliations:** Advanced Materials and Manufacturing (AMM) Research Group, School of Mechanical and Design Engineering, University of Portsmouth, Anglesea Road, Anglesea Building, Portsmouth, Hampshire PO1 3DJ, UK; elwan.le-mener@port.ac.uk (E.L.M.); feldner.marc@gmail.com (M.F.); chulin.jiang@port.ac.uk (C.J.); zhongyi.zhang@port.ac.uk (Z.Z.)

**Keywords:** polymer-matrix composites (PMCs), composite laminates, low-velocity impact, delamination, X-ray micro CT

## Abstract

Understanding the damage mechanisms of composite materials requires detailed mapping of the failure behaviour using reliable techniques. This research focuses on an evaluation of the low-velocity falling weight impact damage behaviour of flax-basalt/vinyl ester (VE) hybrid composites. Incident impact energies under three different energy levels (50, 60, and 70 Joules) were employed to cause complete perforation in order to characterise different impact damage parameters, such as energy absorption characteristics, and damage modes and mechanisms. In addition, the water absorption behaviour of flax and flax basalt hybrid composites and its effects on the impact damage performance were also investigated. All the samples subjected to different incident energies were characterised using non-destructive techniques, such as scanning electron microscopy (SEM) and X-ray computed micro-tomography (πCT), to assess the damage mechanisms of studied flax/VE and flax/basalt/VE hybrid composites. The experimental results showed that the basalt hybrid system had a high impact energy and peak load compared to the flax/VE composite without hybridisation, indicating that a hybrid approach is a promising strategy for enhancing the toughness properties of natural fibre composites. The πCT and SEM images revealed that the failure modes observed for flax and flax basalt hybrid composites were a combination of matrix cracking, delamination, fibre breakage, and fibre pull out.

## 1. Introduction

Over the past decade, consumers’ increased awareness of and expectations towards environmental sustainability, government legislation, an increased sense of corporate social responsibility (CSR) from industry sectors for achieving sustainable development aspirations through a triple bottom line performance (environment, economic, and social), have inspired research on materials which are renewable and recyclable [1,2]. 

Natural fibre-reinforced polymeric composites have been used in a wide range of engineering applications in recent years due to their abundant availability, lower density, and much higher specific strength and modulus than conventional glass and carbon fibre-reinforced composites [3,4,5]. Moreover, these reinforced materials possess a low embodied energy to process and use compared to energy-intensive conventional fibre-reinforced composites. Despite several benefits, there are still some issues which limit the use of natural fibre-reinforced composites in semi-structural and structural applications [6,7]. One of the key issues facing these composites is their hydrophilic nature, which leads to poor fibre matrix interfacial adhesion and lower mechanical properties [8].

Among the natural fibres used in polymeric composites, bast fibres (flax, hemp, jute, and kenaf) stand out as the most promising reinforcements [9,10]. Due to their unique hollow structure, these fibres provide a good damping property, which is very important when it comes to dealing with impact damage and vibration damping behaviours. The good damping properties of bast fibre-reinforced composites make them an attractive alternative to be used in automotive components where impact and damping properties are very important [11,12]. However, their high natural variability, strong affinity to water until saturation, limited processing temperature range, relatively low impact resistance, and low thermal stability negatively influence their long-term durability [13,14]. Moreover, their low impact resistance behaviour under different operation conditions is another concern when these materials are used in automotive and marine sectors.

Mitrevski et al. [15] studied the influence of impactor shape on the impact damage of composite laminates. The results demonstrated that the impactor shape plays a large role in the damage response of composite materials. Composite laminates undergo various impact-induced damage modes under impact loadings. A review carried out by Cantwell and Morton [16] has reported the various impact-induced failure modes of composite laminates. Wisheart and Richardson [17] investigated the impact damage response of complex geometry pultruded glass/polyester composites. 

During the past two decades, there have been many reported works, especially those covering the mechanical and thermal properties of natural bast fibre-reinforced composites. This highlights the significant increase in the demand for these fibres. The automotive sector is leading the way towards using natural bast fibres due to the drives to produce lightweight and sustainable materials and reduce health risks during manufacturing and recycling. Many leading original equipment manufacturers (OEMs) in the automotive sector have been using natural fibre-reinforced composites in various parts, such as door linings, seat cushions, door cladding, and mainly non-structural applications. Nevertheless, due to the lack of sufficient mechanical properties for structural applications, natural fibre-reinforced biocomposites are not fully utilised in semi-structural and structural applications [18,19]. 

In recent years, widespread research has been focused on utilising a hybrid approach which consists of combining two or more reinforcements, in which the synergic effects of both reinforcements are utilised. In order to compensate for the shortcomings of the natural fibre-reinforced composites, glass and carbon fibres, as well as nano particulates, have been used as hybrid constituents [20,21,22,23]. There are many reported works where basalt fibres have been introduced as hybrid reinforcements on natural fibre composites owing to their high thermal stability, good mechanical properties, good corrosion resistance, and natural origin (coming from volcano rock). The work carried out by Dhakal et al. [24] investigated the influence of basalt fibre hybridisation on the post-impact mechanical behaviour of hemp fibre-reinforced composites, and their report suggests that basalt fibre hybrid systems significantly improved the post-impact mechanical properties. Similarly, carbon fibre hybridised flax fibre composites were investigated and it was observed that the hybrid system offered excellent mechanical properties compared to flax fibre non-hybrid composites [25]. Paturel and Dhakal [26] investigated the water absorption and low velocity impact damage characteristics of flax/glass fibre hybrid vinyl ester composites. Their findings suggest that glass fibre hybridised composites significantly reduced the water uptake percentage compared to flax fibre vinyl ester composites without hybridisation. It is evident from the various literature that the impact damage characteristics of natural fibre composites have been well-documented. However, not many studies have been focused on investigating the influence of basalt fibre hybrid flax composites subjected to low-velocity impact loading at different incident energy levels. Additionally, there has been limited work on the influence of hybridisation on the moisture absorption and its effects on the low-velocity impact damage mechanisms. 

This study aimed to investigate the effect of basalt fibre hybridisation on the water absorption and low-velocity falling weight impact behaviour of flax fibre-reinforced vinyl ester hybrid composites with varying incident impact energies. For this, the flax/VE composites were impacted at low impact energies ranging from 50 to 70 Joules, which was sufficient to create impact damage up to penetration. The impact performance of flax and flax/basalt/VE hybrid composites was evaluated in terms of the load bearing capability, energy absorption, and damage modes. In addition, the water absorption behaviour of flax and flax basalt hybrid composites and its effects on the impact damage were also investigated. The damage mechanisms of impacted composite specimens were characterised using non-destructive evaluation techniques, such as X-ray computed micro-tomography (πCT) and scanning electron microscopy (SEM).

## 2. Materials and Methods 

### 2.1. Materials and Laminate Fabrication

**Figure 1:**
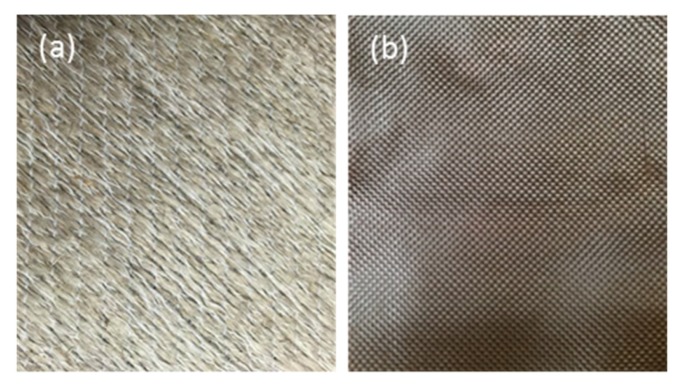
Reinforcements used, (**a**) flax woven fabric, and (**b**) basalt woven fabric.

The matrix material used was vinyl ester, Scott-Bader Crystic VE676-03, obtained from Scott-Bader. Woven flax and woven basalt fibres were used as the reinforcements (±45) as biaxial stitched non-crimp fabrics of 600 g/m^2^. Figure 1 shows the flax and basalt fabric used to make flax and flax/basalt hybrid composites. The chemical and mechanical properties of key bast fibres (flax, kenaf, hemp, and jute), along with basalt fibre included for comparison purposes, are presented in Table 1.

### 2.2. Composite Laminate Fabrication

**Figure 2:**
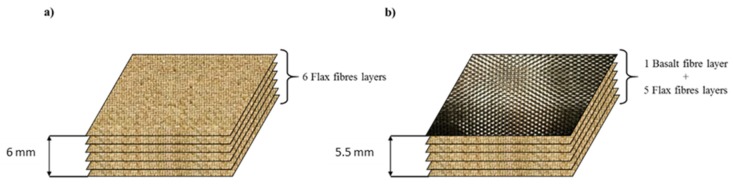
Formulation of laminates: (**a**) Flax/VE laminate with a thickness of 6 mm, and (**b**) flax/basalt/VE hybrid laminates with a thickness of 5.5 mm.

The flax and flax/basalt hybrid laminates were fabricated by the vacuum infusion technique. Two types of samples were fabricated to investigate the influence of hybridisation. In the first set of samples, six layers of flax fibres were used. A second set of samples, including one layer of basalt fibres on the top face and five layers of flax fabric on the rear side, were employed. The main reason for investigating the fibre contents and orientation is to optimise the hybrid materials in the loading direction. The average of the fibre volume fraction of Flax/VE and Flax/Basalt/VE was approximately 31% and 33%, respectively. The void content was approximately 3%. The sample size of a 70 mm by 70 mm square was cut using water jet cutting of the composite panel. Schematics the of flax and flax basalt hybrid composite laminates are shown in Figure 2. 

### 2.3. Moisture Absorption Measurement

The moisture uptake behaviour of flax/VE and flax/VE/basalt hybrid laminates was investigated in accordance with BS EN ISO:1999 [31]. Five specimens, consisting of 70 mm by 70 mm squares of flax/VE and flax/VE/basalt hybrid composites, were placed in a desiccator for 48 h and weighted near to 0.1 mg. Then, the specimens were immersed in de-ionised water at room temperature. After 24 h of immersion, the specimens were taken out and the surface was dried with absorbent paper. The process was repeated until the saturation moisture was reached. The percentage of moisture uptake was calculated using Equation (1):

(1) $$M(\%)=\frac{M_{t}{-M}_{0}}{M_{0}}\times 100$$

where *M* (%) is the moisture uptake in percentage, *M*_t_ is the weight of the water-immersed specimen at a given time, and *M*_0_ is the initial mass of the specimen in a dry condition. 

### 2.4. Low-Velocity Drop Weight Impact Testing

Low-velocity instrumented falling weight impact testing was conducted by using an instrumented falling weight impact testing (IFWIT) machine. A Zwick/Roell impact test machine (IFW 413) was used for the testing in accordance with the British Standard BSEN ISO 6603-2 recommendations [32]. The hemispherical impact tup used was made of steel and had a 20 mm diameter. The incident energy was tailored by adjusting the release height of the impact mass, i.e., changing the impact velocities (while keeping the other parameters constant). To analyse the damage behaviour of flax and flax/basalt hybrid vinyl ester hybrid composites, the three different incident energies employed were 50, 60, and 70 Joules, respectively (corresponding impact velocities of 2.08, 2.28, and 2.46 m/s). The test specimens were 70 mm by 70 mm squares.

### 2.5. X-Ray Computed Micro-Tomography (πCT)

πCT, XT H 225 was used to assess the barely visible impact damage failure. The samples were subjected to the three different incident energies of 50, 60, and 70 Joules and examined using X-ray (πCT) to effectively evaluate the extent of damage due to impact loadings.

### 2.6. Scanning Electron Microscopy (SEM)

In order to investigate the damage mechanisms of the impacted samples, the surfaces of dried specimens and room temperature immersed specimens were examined using SEM Zeiss EVO LS10. Before examination, the samples were placed in a desiccator to remove all of the water of the samples, in order to avoid evaporation during characterisation. The specimens were also surface prepared and the damaged area was imaged. 

## 3. Results and Discussion 

### 3.1. Flax/Basalt/VE Hybrid Composite Lay Up

**Figure 3:**
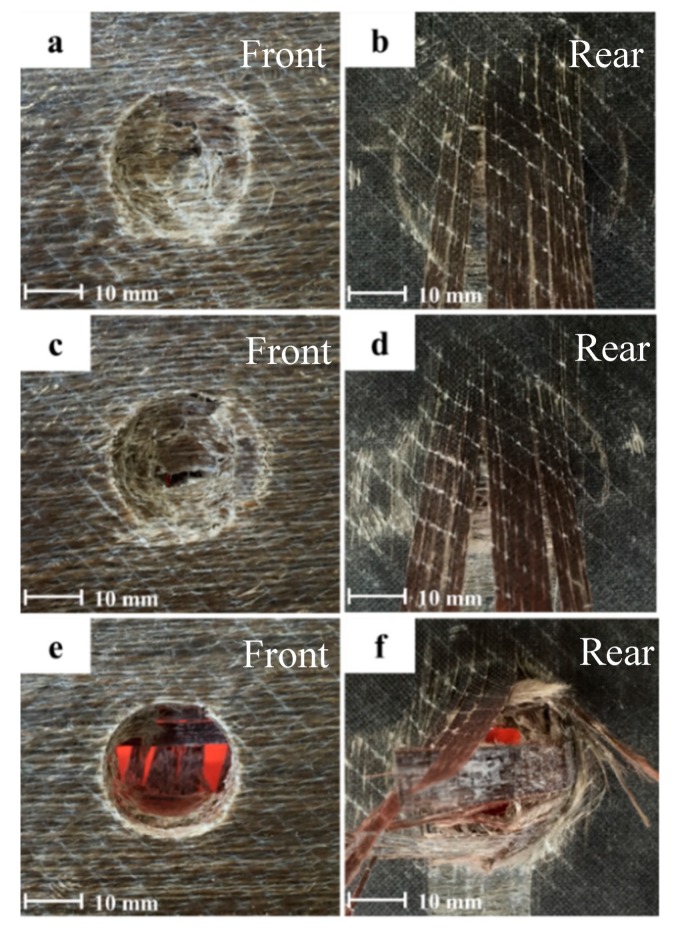
Images of damage progression on the front and rear faces of a flax/VE/basalt hybrid composite where basalt was placed on the rear side of the composite panels impacted in the range of (**a**,**b**) 50 J, (**c**,**d**) 60 J, and (**e**,**f**) 70 J.

One of the main aims of this study was to find out if hybridising one side of the composite plate would provide optimal hybrid effects. Figure 3 shows the hybrid sample with a rear basalt layer. It is clear from the figure that the basalt layer exhibits push-out delamination. As the incident energy increases, the delamination also increases. Delamination is one of the most prevalent failure mechanisms in composite laminates. This phenomenon becomes even greater when two different types of fibres are hybridised. It can be seen that the impactor has perforated and all the flax layers have fractured, but the basalt layer has not fractured; these impacted images show delamination of the basalt layer.

**Figure 4:**
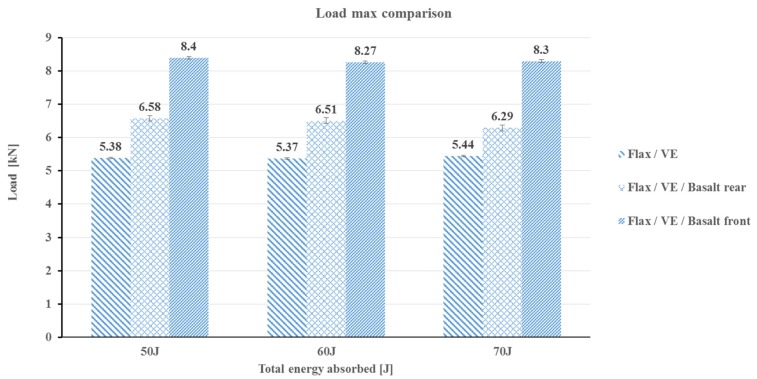
Maximum load comparison of different impact configurations of flax/VE, flax/VE/basalt rear, and flax/VE/basalt front for each incident energy employed.

The influence of the basalt fibre layer on the front and rear side of the laminates is further explain in Figure 4. It is evident from the figure that flax composites with a basalt layer on the top impact face provided optimal properties in comparison to the basalt fibre on the rear side under the three different incident impact energies of 50, 60, and 70 Joules. The main reason for this phenomenon is that when basalt fibre was placed on the rear side of the laminates, a significant amount of delamination was observed, which is shown in Figure 4. Employing this evidence, the remaining investigation was carried out on samples where basalt fibre was placed on the top side of the flax/VE composites. Just placing one layer of basalt fibre on the top of the flax/VE composite laminate provides a good design choice to fabricate high-performance composite laminates using a simple and cost-effective method. 

### 3.2. Impact Damage Characteristics

#### Load and Energy Absorption Capabilities 

Important impact parameters and corresponding values obtained from the low-velocity testing for flax and flax basalt hybrid samples at three different incident impact energies (50, 60, and 70 Joules) are presented in Table 2.

**Figure 5:**
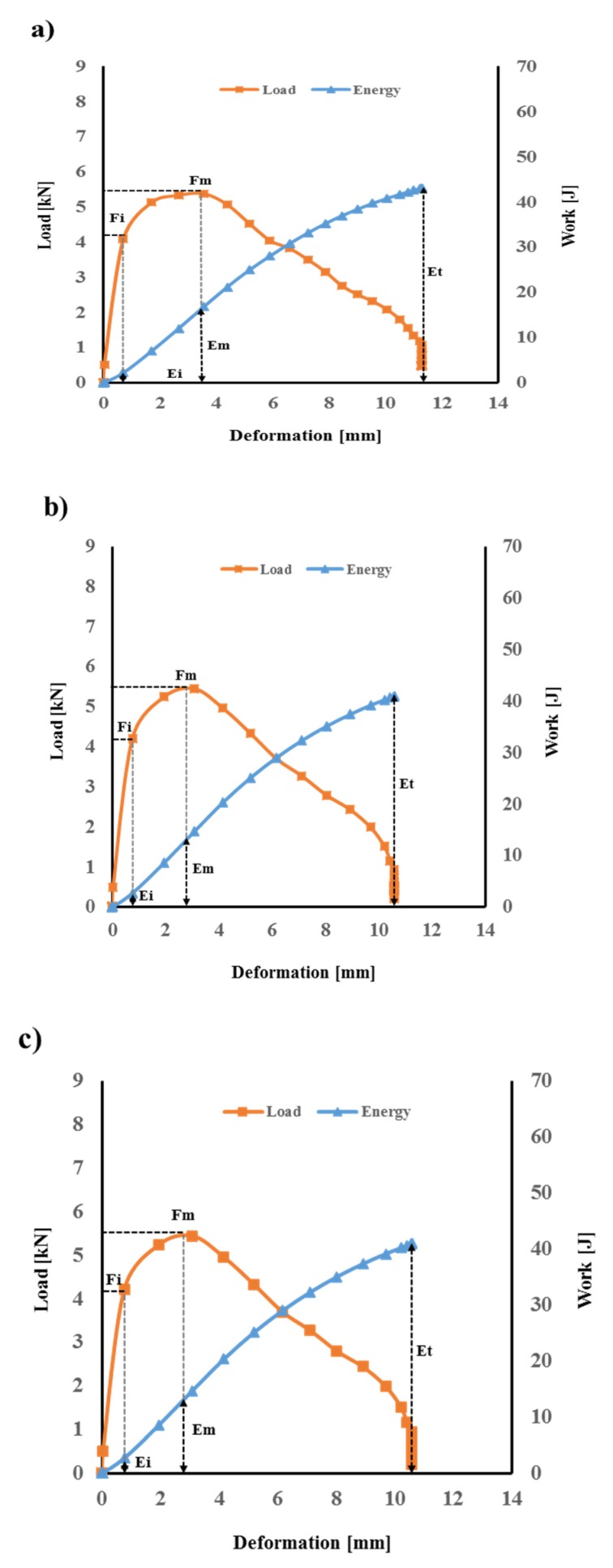
Load and work vs. deformation for flax/VE composites: (**a**) flax 50 J, (**b**) flax 60 J, and (**c**) flax 70 J. FM, load maximum; FI, incipient damage load; EM, energy maximum; EI, incipient damage energy; ET, energy total.

Load–deformation–energy traces obtained from the impact testing for flax/VE composites are shown in Figure 5. 

Two of the most used parameters to assess damage resistance in composites after an impact are the impact energy and absorbed energy. The impact energy represents the maximum energy that the specimen can transform (it is equal to the kinetic energy of the impactor right before dart contact with the sample when the impact takes place), whereas the absorbed energy is the unrecoverable energy dissipated by the system (including energy dissipated by friction and, most importantly, by mechanisms which are peculiar to the material).

**Figure 6:**
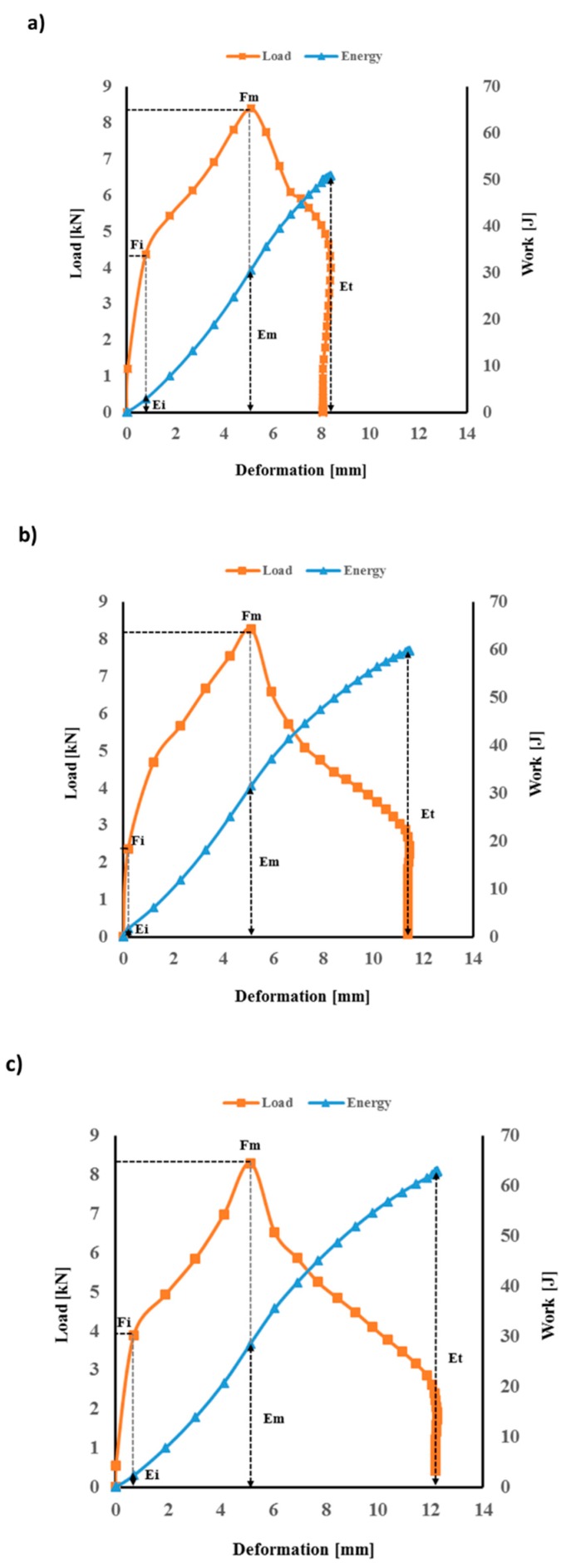
Load and work vs. deformation for flax/VE/basalt hybrid composites: (**a**) flax 50 J, (**b**) flax 60 J, and (**c**) flax 70 J. FM, load maximum; FI, incipient damage load; EM, energy maximum; EI, incipient damage energy; ET, energy total.

**Figure 7:**
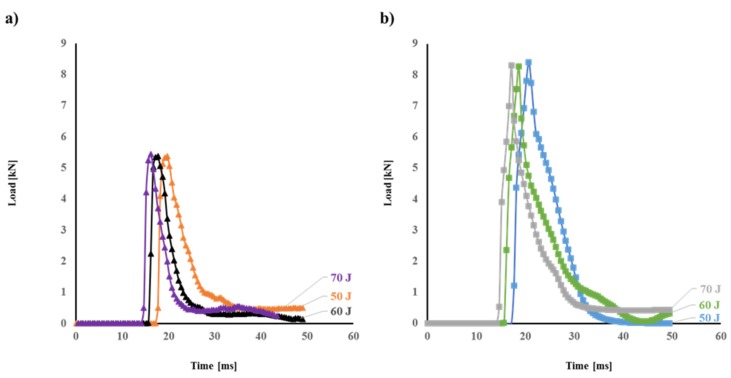
Typical load vs. time traces for 50 J, 60 J, and 70 J impacted (**a**) flax and (**b**) flax/basalt hybrid composites.

The absorbed energy can be calculated from load vs. deformation curves. In order to evaluate the laminate’s performances, the transient response of each laminate was recorded in terms of the load, energy, and displacement. It can be observed from Figure 5 and Figure 6 that the peak contact force is higher for the flax/basalt hybrid composite than that of flax/VE without hybridisation, which indicates that the hybrid specimens offer a higher impact resistance during impact events. A similar positive hybrid effect can be observed in load–time traces (Figure 7).

The load–deformation–energy traces for flax/basalt/VE hybrid composites are depicted in Figure 6. Flax/VE/basalt hybrid composites absorb more energy than flax/VE, as illustrated in Figure 6. We can observe similar curves for all three energy levels for flax/basalt hybrid composites. It is evident from Figure 6 that the applied incident energies (50, 60, and 70 Joules) were not high enough to penetrate or perforate (showing rebound energy) the hybrid samples, indicating their superior mechanical behaviour and higher energy dissipation potential compared to flax/VE samples without hybridisation. It is clear that flax/basalt hybrid composites exhibited a significantly improved impact performance compared to flax/VE composites with hybridisation.

Figure 6 illustrates the performance of flax basalt hybrid composites in terms of representing different impact parameters. It is clear from the results that flax/basalt hybrid composites exhibited significantly improved impact performances compared to flax/VE composites without hybridisation (Figure 5). It can also be observed that basalt hybridisation contributes to increasing the deformation of composites. This improvement could be attributed to the higher failure strain of basalt fibres compared to commonly used natural fibres such as flax and hemp. This phenomenon provides a balanced property, as one would expect for hybrid systems. These results indicate that basalt fibre hybridisation into natural fibre composites provides a promising strategy for enhancing the overall impact toughness. Such hybrid effects have been reported for improved mechanical properties, such as tensile, flexural, and fracture toughness behaviours [33,34].

Figure 7 shows the load–time traces of impacted flax and flax/basalt hybrid composite specimens. In this case, the peak load is the same as previously shown (Figure 5 and Figure 6). However, the test time required to complete the impact event is important to consider. The time the striker was in contact with the impacted specimens is approximately the same for each composite. However, the time taken to complete the impact event is longer for flax/basalt hybrid composites compared to flax/VE composites. This is an indication that hybrid composites have better impact resistance behaviour as a result of the hybrid effect.

### 3.3. Moisture Absorption Behaviour

**Figure 8:**
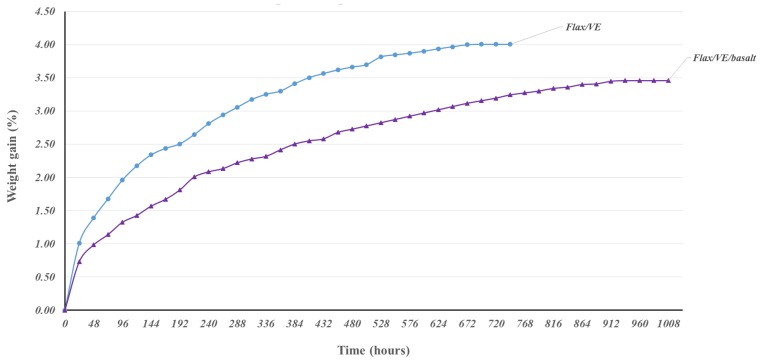
Water absorption comparison of flax/VE and flax/VE/basalt hybrid composites.

Figure 8 depicts the moisture absorption curves of flax/VE and flax/basalt/VE hybrid composites. It is evident from the curves that the moisture uptake at the beginning is linear and rapidly increases for flax/VE composites compared to flax/basalt hybrid composites. After the initial rapid rise, the moisture uptake slows down and reaches saturation at 768 h (30 days) for flax composites, whereas it takes longer—1008 h (42 days)—for flax/basalt hybrid composites. The longer time taken for hybrid composites to reach saturation moisture absorption can be attributed to the influence of basalt fibres restricting the flow of water molecules, as basalt fibres have better water repellence behaviour compared to flax/VE composites. Nonetheless, for the side where only flax is exposed, there would still be moisture ingress taking place at a higher rate than where basalt fabric was placed as a hybrid layer. This can be observed by the moisture uptake percentage difference between flax and flax/basalt hybrid composites, which is only 0.5%. If the basalt fabric was placed on both sides of the flax samples, the moisture uptake percentage of hybrid composites would have been far lower. Moreover, the sides of the both types of composites were not sealed, which is another reason for the higher moisture absorption displayed by both composites.

The maximum weight gain reported for vinyl ester matrix is 1.07% at room temperature [30]. The maximum weight gain percentages for flax/VE and flax/basalt/VE hybrid composites were approximately 4% and 3.5%, respectively. The lower moisture uptake percentage for flax/basalt hybrid composites is attributed to the barrier effects of top-layer basalt fibre on flax/VE composites. The moisture absorption behaviour for both composites indicates Fickian behaviour, which is rapid in the beginning and slowly reaches saturation. 

By comparing the flax-basalt hybrid specimens with those made entirely of flax, it can be seen that the addition of basalt fibre improves the moisture absorption resistance of the hybrid composite. Since the five layers of flax fibre were sandwiched by one ply of basalt fibre, the total area of flax exposed to the water was decreased for flax/VE composites. The reason for the difference in moisture absorption between the flax and basalt hybrid specimens can be further explained by considering the chemical composition of flax fibres. The cellulose in the flax fibre is what provides the majority of the stiffness and strength; however, the semi-crystalline structure contains a large amount of hydroxyl groups, which give the fibre hydrophilic characteristics. By covering flax fibres with basalt fibres in a hybrid composite, the surface area exposed to water is reduced and therefore absorbs less moisture. 

#### Influence of Moisture Absorption on the Impact Resistance Behaviour 

**Figure 9:**
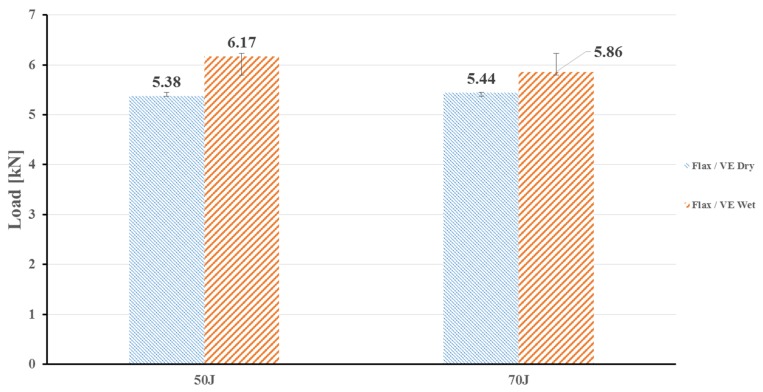
Impact load at different energy levels for flax/VE composites in dry and wet conditions.

The influence of moisture absorption on the flax composite in dry and wet conditions was investigated. The effects of moisture absorption on the load bearing capability of flax/VE composites impacted at two different incident energy levels are shown in Figure 9. The wet flax/VE specimens displayed a higher peak load compared to wet specimens, slightly outperforming the dry sample. This could be attributed to water absorption-induced plasticisation of the vinyl ester matrix leading to an increase of deformation and impact energy absorption [5,23]. 

Generally, when natural fibres absorb moisture, they swell, which promotes the development of adverse effects on the mechanical properties, such as tensile, flexural, and fatigue properties, due to the weak fibre matrix interface. However, as far as the impact performance is concerned, the results from this study suggest that there was no negative influence of moisture absorption on the load. Instead, the wet samples withstood a slightly higher peak load than the dry ones. This could be attributed to engrossed amounts of water causing swelling of the flax fibres, which could fill the gaps between the fibre and vinyl ester matrix and could have eventually led to an increase of impact load [5]. 

### 3.4. Damage Characterisation 

#### 3.4.1. Damage Behaviour in Dry Conditions

**Figure 10:**
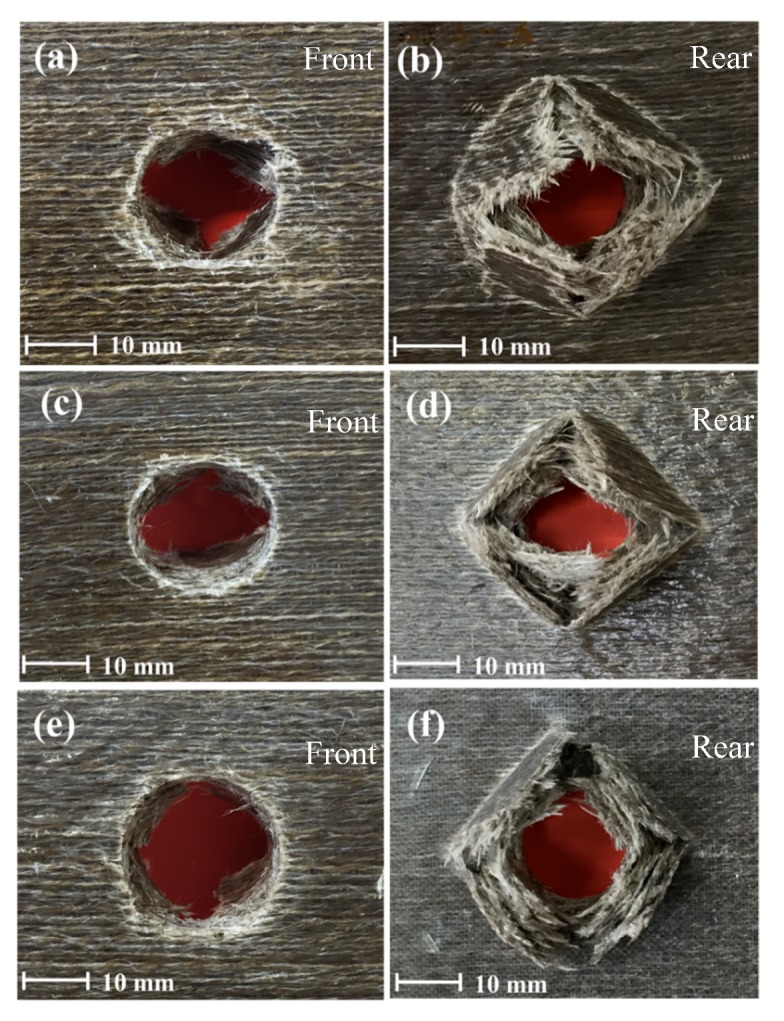
Images of damage progression on front and rear faces of flax/VE composite samples impacted in the range of (**a**,**b**) 50 J, (**c**,**d**) 60 J, and (**e**,**f**) 70 J.

Figure 10 depicts the damage of front and rear faces of impacted flax/VE composite specimens. As can be clearly observed, all the samples impacted at 50, 60, and 70 Joules were fully penetrated. The incident energy of 50 Joules was enough to cause damage to these groups of samples. 

**Figure 11:**
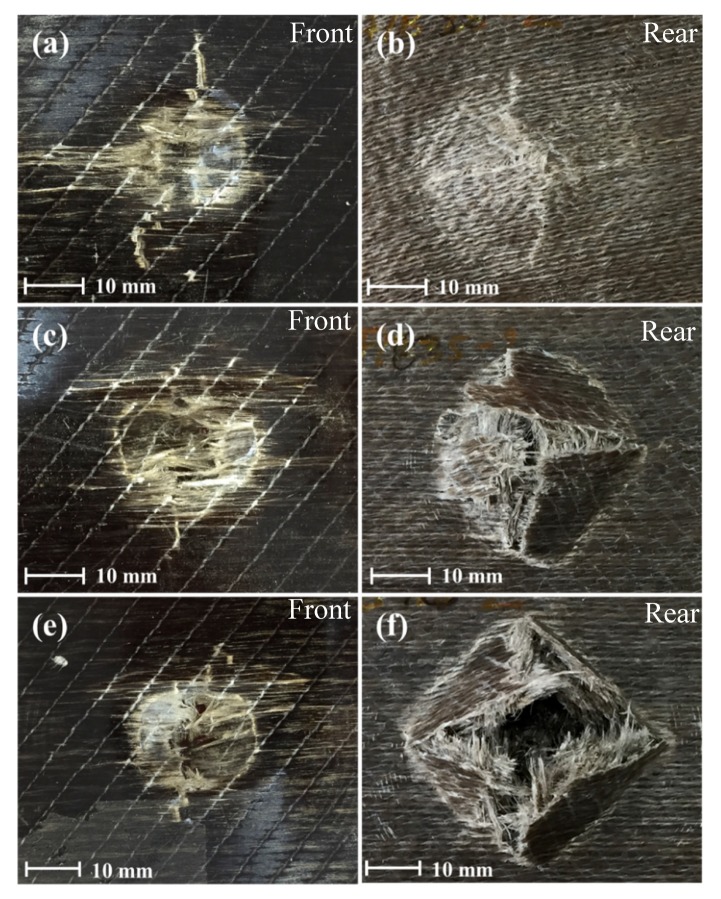
Images of damage progression on front and rear faces of flax/VE/basalt hybrid composite panels impacted in the range of (**a**,**b**) 50 J, (**c**,**d**) 60 J, and (**e**,**f**) 70 J.

Figure 11 shows the damage of the hybrid sample with one basalt layer on the top. In the pictures (a) and (b), a 50 J incident energy was not high enough to perforate the sample thanks to the basalt layer on the top. However, in the other images (Figure 11e–f) for 60 and 70 Joules of incident energy, the maximum energy absorbed is exceeded and all the layers of fibres are broken. As a result, the samples are fully penetrated. 

#### 3.4.2. Visual Observations of Damage Behaviour in Wet Conditions 

**Figure 12:**
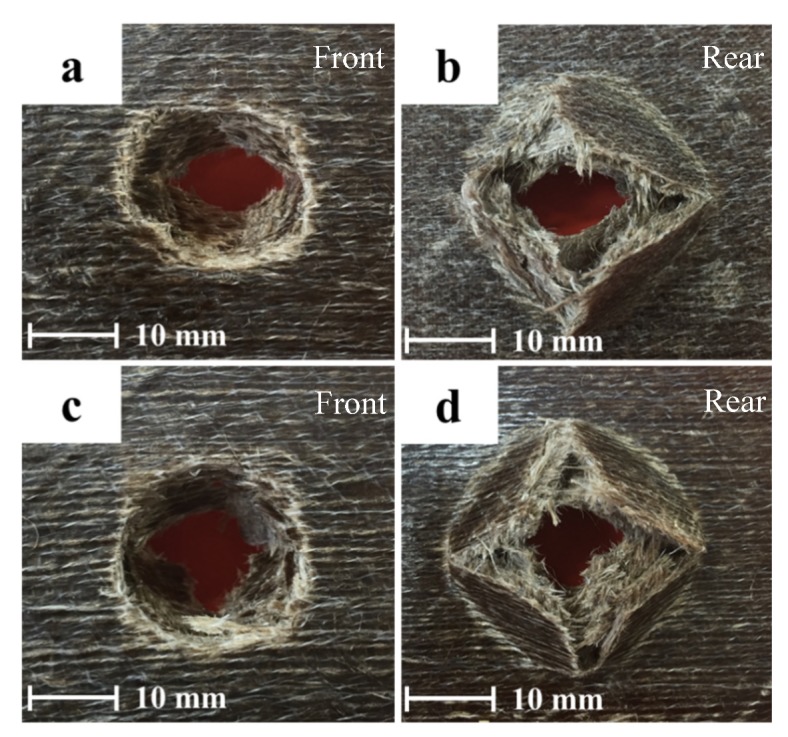
Images of damage progression on front and rear faces of flax/VE composite panels impacted in the range of (**a**,**b**) 50 J and (**c**,**d**) 70 J after water absorption.

Figure 12 shows impacted front and rear specimens for water-immersed flax/VE samples impacted at 50 and 70 Joules of incident energies. It can be observed that the wet samples do not have as clear holes as those of dry specimens (Figure 10), indicating the increased ductility of water-immersed samples. 

#### 3.4.3. Damage Behaviour from SEM Observations

**Figure 13:**
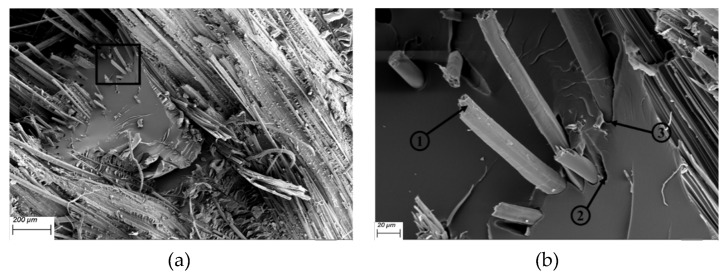

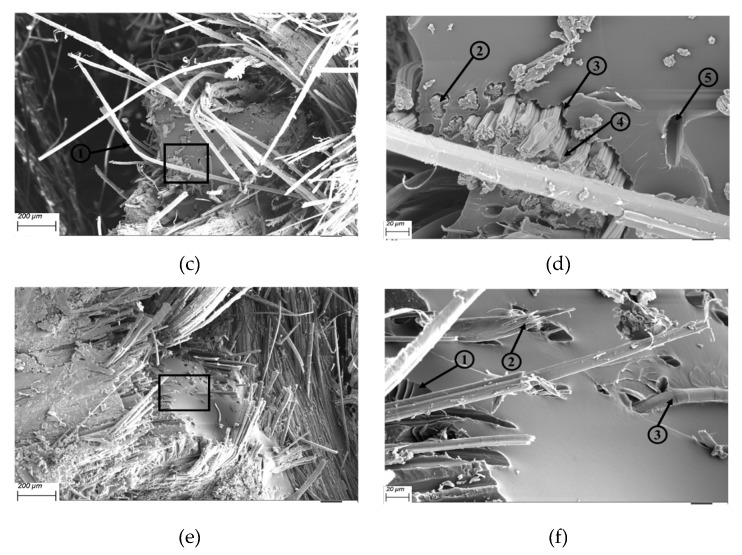
SEM images of flax/VE composites: (**a**) 150 (**b**) 1000× magnification with 50 J, (**c**) 150× magnification with 60 J, magnification with 50 J, (**b**) 1000× magnification with 50 J, (**c**) 150× magnification with 60 J, (**d**) 1000× magnification with 60 J, (**e**) 150× magnification with 70 J, and (**f**) 1000× magnification with 70 J.

**Figure 14:**
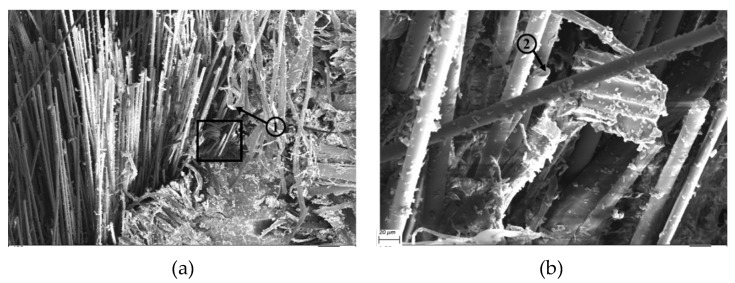

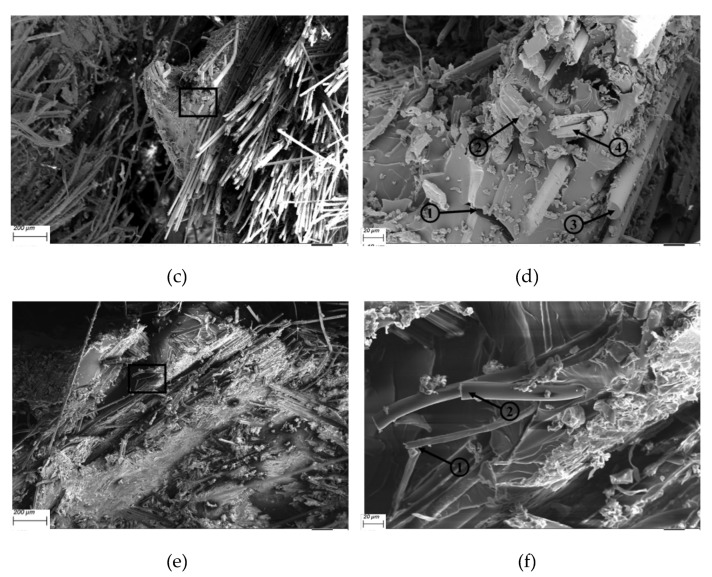
SEM images of flax/VE/basalt hybrid composites: (**a**) 150× magnification with 50 J, (**b**) 1000× magnification with 50 J, (**c**) 150× magnification with 60 J, (**d**) 1000× magnification with 60 J, (**e**) 150× magnification with 70 J, and (**f**) 1000× magnification with 70 J.

**Figure 15:**
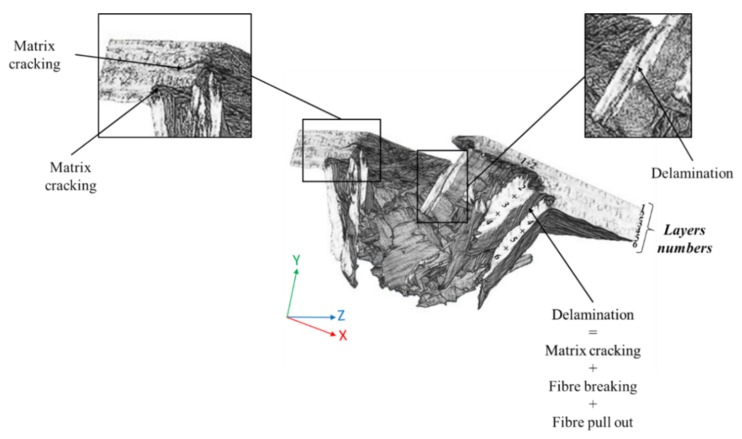
Micro CT scan picture of a flax composite specimen (3/4 of the impact hole) after an impact test, with a 50 J impact energy, showing different failure modes in the specimen.

SEM images illustrated in Figure 13 and Figure 14 reveal matrix cracking, fibre fracture, and delamination on the fractured surfaces of impacted flax/VE and flax/basalt/VE hybrid samples. These damages are in close agreement with those observed in previous studies on flax/carbon epoxy and flax/glass vinyl ester matrix hybrid composites [25,26]. The SEM images further reveal that the extent of damage increased with the increase of the incident energy level. These images further suggest that at a higher energy level, the composites undergo severe damage, with evidence of fibre breakage and pull out, especially in the case of flax/VE composites (Figure 13). For flax/basalt/VE hybrid composites, delamination and fibre bending can be observed (Figure 14). It is also worth mentioning that with basalt fibre on the top layer of hybrid composites, the energy dissipation is increased, which allows an enhancement in impact and fracture toughness behaviours [35,36]. Moreover, it can be observed that for higher energy-impacted samples, more severe fibre damage and breakage can be observed. Different failure modes for flax/VE composites are further explained by the Micro-CT scan illustrated in Figure 15, which compliments the observation made via SEM images. From the annotation provided for the SEM images of flax/VE composites, as shown in Figure 13a–d, the following can be observed:

(1) Fibre breakage; (2) delamination; (3) fibre debonding; and (4 and 5) fibre pull out. 

From the annotation provided for the SEM images of flax/basalt/VE hybrid composites, as shown in Figure 14a–d, the following can also be observed:

(1) Matrix cracking and fibre bending and (2, 3, and 4) basalt fibre breakage and fracture. 

**Figure 16:**
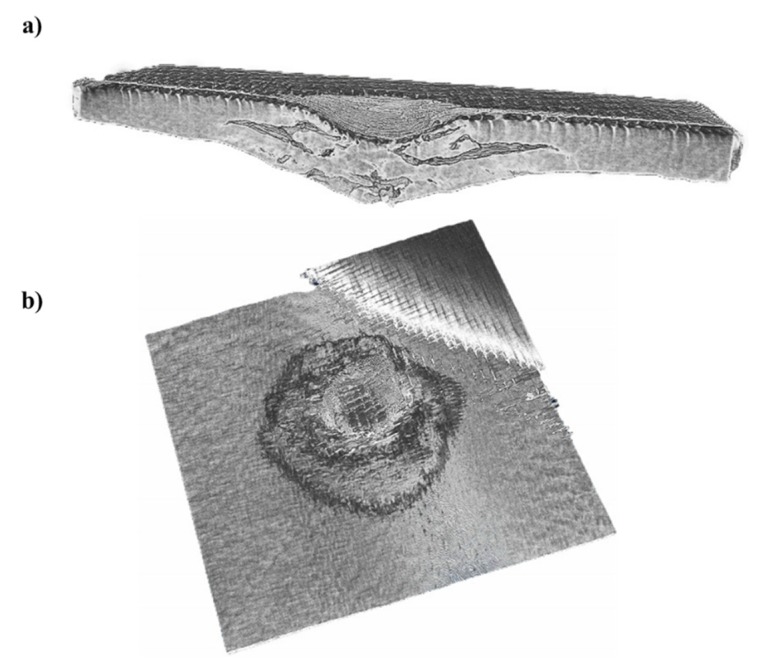
Micro CT scan pictures of a flax/basalt hybrid composite after an impact test, with a 50 J impact energy: (**a**) half-view of the specimen with impact failure, in the form of delamination, and (**b**) top view of the damaged area’s shape.

Figure 16 shows a 3D view of the impacted flax/basalt hybrid sample. Unlike the image illustrated in Figure 15 for flax/VE without hybridisation, the hybrid sample shows less damage and different failure modes, such as delamination.

**Figure 17:**
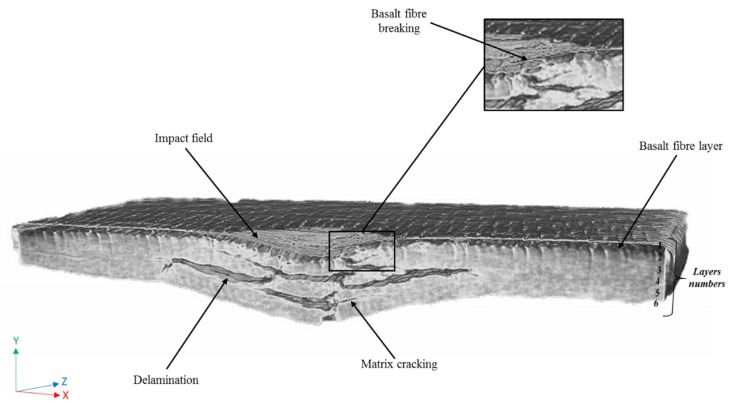
Micro CT scan picture of a flax/basalt hybrid composite specimen (half-view) after an impact test, with a 50 J impact energy, with different failures displayed by the specimen.

Figure 17 illustrates a 3D half-view of a hybrid composite. It can be clearly observed that the basalt hybridised sample exhibited larger delamination within the top and adjacent layer. In comparison to the flax/VE composite, the flax/VE/basalt hybrid composite does not display penetration. Indeed, the basalt layer has absorbed a higher impact energy.

## 4. Conclusions

The effects of basalt fibre hybridisation on the low-velocity falling weight impact behaviour of flax/VE bio-based composites have been investigated following water immersion at room temperature at three incident impact energies: 50, 60, and 70 Joules. The results show that the incident energy has a significant influence on the load bearing capability and total energy absorption characteristics. For a short period of water immersion, it was found that water immersion did not result in a reduction in the impact load and energy absorption. Additionally, the basalt fibre hybridisation at the front side of the laminates significantly enhanced the impact load and total energy of flax/VE composites, showing the potential of flax/VE bio-based composites for semi-structural or structural applications. The damage mechanisms following the X-ray micro CT examination and SEM characterisations performed reveal the greater resistance to penetration and perforation by basalt hybrid composites, which is an indication of their higher impact performance, offering balanced properties of environmental benefits and enhanced impact behaviour. The damage modes for flax/VE composites were matrix cracking, fibre breakage, and fibre pull out. Comparatively, for the flax/basalt hybrid composites, the predominant failure mode was matrix cracking and delamination.

## Figures and Tables

**Table 1:** Chemical composition, and physical and mechanical properties of commonly used natural bast fibres [27,28,29,30].

Fibres	Cellulose	Hemi-Cellulose	Lignin	Pectin	Density (g/cm^3^)	Tensile Strength (MPa)	Young’s Modulus (GPa)	Failure Strain (%)
Flax	70.5	16.5	2.5	0.9	1.45	700	60	2.3
Kenaf	78.5	8–13	21.5	0.6	1.40	350–600	21–60	1.6–3.5
Hemp	81	20	4	0.9	1.48	530	45	3
Jute	67	16	9	0.2	1.40	325	37.5	2.5
Basalt *	-	-	-	-	2.70	4800	90	3.15
E-glass *	-	-	-	-	2.55	3400	72	3.4
VE matrix	-	-	-	-		70	3.5	0.02

* For comparison purposes.

**Table 2:** Important impact parameters and corresponding values obtained from impact testing.

Specimen	Peak Load, Fm (kN)	Incipient Damage Load, Fi (kN)	Maximum Energy, Em (J)	Incipient Energy, Ei (J)	Total Energy, Et (J)
Energy at 50 J					
Flax/VE	5.39	4.09	16.81	2.22	43.03
Flax/VE/basalt	8.40	2.36	31.67	1.67	59.88
Energy at 60 J					
Flax/VE	5.42	4.11	14.67	2.70	40.44
Flax/VE/basalt	8.27	2.36	31.67	1.67	59.88
Energy at 70 J					
Flax/VE	5.51	4.21	15.73	2.82	41.44
Flax/VE/basalt	8.30	3.90	28.58	2.34	62.89
